# Headcount and FTE data in the European health workforce monitoring and planning process

**DOI:** 10.1186/s12960-016-0139-2

**Published:** 2016-07-16

**Authors:** Edmond Girasek, Eszter Kovács, Zoltán Aszalós, Edit Eke, Károly Ragány, Réka Kovács, Zoltán Cserháti, Miklós Szócska

**Affiliations:** Health Services Management Training Centre, Semmelweis University, Kútvölgyi út 2, Budapest, Hungary

**Keywords:** Human resources for health, Headcount, FTE, Joint Questionnaire, Health workforce planning

## Abstract

**Background:**

Health workforce (HWF) planning and monitoring processes face challenges regarding data and appropriate indicators. One such area fraught with difficulties is labour activity and, more specifically, defining headcount and full-time equivalent (FTE). This study aims to review national practices in FTE calculation formulas for selected EU Member States (MS).

**Methods:**

The research was conducted as a part of the Joint Action on European Health Workforce Planning and Forecasting. Definitions, categories and terms concerning the five sectoral professions were examined in 14 MS by conducting a survey. To gain a deeper understanding of the international data-reporting processes (Joint Questionnaire on Non-Monetary Health Care Statistics—JQ), six international expert interviews were conducted by using a semi-structured interview guide.

**Results:**

Of the 14 investigated countries, four MS indicated that they report FTE to the JQ and that they also calculate FTE data for national planning purposes. The other countries do not use FTE data for national purposes, but most of them do use special calculations and/or estimation methods for converting headcount to FTE. The findings revealed significant differences between national calculation methods when reporting FTE data to the JQ. This diversity in terms of calculations and estimations can lead to biases with respect to international comparisons. This finding was reinforced by the expert interviews, since the experts agreed that the activities of healthcare professionals are a fundamental factor in HWF monitoring and planning. Experts underscored that activity should also be measured by FTE, and not only by headcount.

**Conclusions:**

FTE and headcount are significant factors in HWF planning and monitoring; therefore, national data collections should place emphasis on collecting data and calculating the appropriate indicators. National FTE could serve as a call to action for HWF planners due to the lack of matching international FTE data.

At the international level, it is beneficial to monitor the trends and numbers regarding human resources and working time. For the moment, the exchange of information and mutual assistance for developing the capacity to apply common methodology could be a first step towards the standardisation of data collections.

**Electronic supplementary material:**

The online version of this article (doi:10.1186/s12960-016-0139-2) contains supplementary material, which is available to authorized users.

## Background

Healthcare is a labour-intensive sector; therefore, the EU active population engaged in the healthcare sector is one of the largest [1]. The delivery of healthcare services highly depends on possessing an appropriate number of skilled health personnel [2]. The impact of demographical, epidemiological and social/societal changes indicates increasing care needs for the population [3]. Only flexible health systems and health workforces can adapt efficiently to this continuous and turbulent change [3].

The aim of health workforce monitoring and planning is to ensure the appropriate number and type of health human resources, in order to deliver the right services to the right people at the right time [4]. Evidence-based information is needed to simultaneously implement appropriate policy-making at the national and EU levels [5].

Health workforce monitoring is an emerging issue intended to support decision-making at both aforementioned levels [6]. Recognising its importance, the health workforce crisis was placed onto the agenda of the European Health Action Plan and the EU Health Programme. Furthermore, the European Commission (EC) launched the Joint Action on European Health Workforce Planning and Forecasting (JA EUHWF 20122201), which brings together partners representing countries, regions and interest groups from throughout Europe and beyond, including non-EU countries and international organisations. The Joint Action aims to improve the capacity for health workforce planning and forecasting by supporting European collaboration.

### The role of FTE calculations in national health workforce planning

Health workforce (HWF) monitoring and planning analyses the current HWF situation and contributes to exploring future challenges. Calculations provide data on the density of human resources and tend to show the imbalances of a given territory. Headcount measures the stock of healthcare professionals available for delivering healthcare services. The headcount number of health professionals does not factor in working hours (part-time work or actual working hours) or holidays, which may differ between countries and professionals. Therefore, headcount can only reveal the maximum available capacity of a given healthcare system.

While headcount currently seems to be the most widely collected data category, full-time equivalent (FTE) is used to measure employed persons in a way that makes them comparable, although they may work a different number of hours per week [6, 7]. The simplest calculation of FTE may be done by adjusting the headcount numbers by part-timers (regardless of their actual working hours) or by working hours. In order to conduct health workforce monitoring and planning, it is necessary to determine the volume of available resources at the national level, which can support the estimation of the efficiency and productivity.

### FTE at the international level

In the European Union, health workforce planning activities are conducted under the authority of the Member States. Nonetheless, an international comparison of headcount data (the number of currently practising professionals) and FTE (adjusted by working hours) is beneficial for understanding the different contexts of the health workforce across countries [8]. In spite of the importance of the FTE indicator, international agreement on its method of calculation and utilisation is currently lacking [9].

In order to enhance cross-national comparability, data collection efforts should be processed by using internationally standardised classifications with the greatest level of detail possible [10]. The Joint Questionnaire on Non-Monetary Health Care Statistics (JQ) might serve as a tool in the process of harmonising data sources and data collection guidelines concerning the numbers and composition of health professionals [6, 11]. The JQ is a joint initiative of the OECD, EU and WHO [6], which aims to highlight the data that is to be collected in a standardised way, in order to have a comparison-oriented data set for health workforce-monitoring purposes in the EU. The JQ collects headcount and FTE data about each of the five sectoral professions.

The OECD elaborated three methods for FTE calculation, thereby aiming to encourage countries to use standard formulas in order to enhance cross-national comparability. The OECD recommends the following three methods for FTE calculation [11]:

- Actual/usual working hours: the number of hours actually worked, divided by the average number of hours worked in full-time jobs (e.g. 50 h actually worked by a doctor/40 h per week as a full-time job = 1.25 FTE)
- Contractual working hours: a worker with a full-time contract = 1 FTE. The number of hours of work mentioned in a contract divided by the normal number of hours worked in full-time jobs
- In cases where there is a lack of information on working hours: a worker with a full-time contract = 1 FTE and two part-time workers = 1 FTE

Based on the results of the data collected by the JQ, the OECD released a summary table (Table 1) about the number of countries that supply data to the JQ on hospital employment in headcount and FTE [12]. Table 1 demonstrates notable gaps concerning the availability of FTE data in regard to health professionals. The JQ collects FTE data only in one specific category: active employment in hospitals.

**Table 1:** Number of countries supplying data on headcounts and FTEs for hospital employment (JQ 2011) [12]

	Number of persons (head counts)	Full-time (FTE) equivalent persons
Total hospital employment	22	18
Physicians employed in hospitals	23	18
Professional nurses and midwives employed in hospitals	25	17
Associate nurses employed in hospitals	21	15
Healthcare assistants employed in hospitals	18	15
Other health service providers employed in hospitals	22	18
Other staff employed in hospitals	19	16

This paper aims to review national FTE calculations and data collections. It attempts to present selected countries that currently apply the FTE calculations required by the JQ data collection. The present paper does not intend to discuss other national FTE calculations that are not covered by the JQ.

## Methods

The research was conducted as a part of the Joint Action on European Health Workforce Planning and Forecasting (JA EUHWF 20122201) project. In the first phase, the EC approached each Member State and the participating countries designated a national entity for the project. The research results presented in this paper are based on the activities conducted in the “Terminology gap analysis” of Work Package 4 (WP4) “Data for improved health workforce planning”.

### Survey

A questionnaire survey was conducted in 2013 to explore and clarify the details of the OECD-WHO-Eurostat Joint Questionnaire (JQ) [11] data-reporting process. Definitions/categories/terms concerning the five sectoral professions: medical doctors, nurses, dentists, midwives and pharmacists [13] and data sources used in different countries, were examined. The survey aimed to map the difficulties at the Member State (MS) level to conduct a terminology gap analysis. By gaining a thorough understanding of the national data supplied to the JQ, recommendations for an improved data collection process might be presented in the future.

The survey was developed by the Health Services Management Training Centre, Semmelweis University, the leader of WP4’s “Data for improved health workforce planning” activity. According to the Grant Agreement—which described the covered topic—the draft questionnaire was compiled. Consultations and discussions were carried out during the development of the survey, and three collaborating institutions from different countries pilot-tested it. During these consultation processes, the content of the questionnaire was fine-tuned and finalised.

The survey focused on various terminology-related topics: health workforce terminology, definitions and the content of the different data categories covered in the JQ (including labour activity, headcount and FTE issues), data collection practices and health workforce mobility data collection issues (the questionnaire survey items related to FTE can be read in Additional file 1). The questionnaire has one major limitation: it cannot differentiate between the FTE calculations between professions.

The English language survey was distributed in digital format (an MS Word file) to the associate partners of this research activity in 14 countries (Belgium, Cyprus, Finland, Germany, Greece, Hungary, Iceland, Ireland, Italy, Poland, Portugal, Spain, the Netherlands and the United Kingdom). The countries were represented by an authority or research institute, which were previously designated by the respective Ministries of Health (Table 2). After completing the survey, some problematic issues were discussed and clarified with the country representatives.

**Table 2:** Respondent organisations by country

Belgium	Federal Public Service Health
Cyprus	Ministry of Health
Finland	The National Agency for Health and Welfare
Germany	University of Bremen
Greece	National School of Public Health
Hungary	Semmelweis University Health Services Management Training Centre
Iceland	Ministry of Welfare
Ireland	Department of Health
Italy	Agenas, National Institute for Regional Healthcare
Poland	Ministry of Health
Portugal	Ministry of Health
Spain	Ministry of Health
The Netherlands	Capaciteitsorgan/NIVEL
The United Kingdom	Centre for Workforce Intelligence

Each of the contacted organisations completed the survey, so that in total, 14 country responses were taken into consideration for further analysis. The survey data were processed into an analytical database.

### Expert interviews

To gain a deeper understanding of the reporting process for the JQ, expert interviews were conducted by using a semi-structured interview guide. The guide was prepared by the research team of the Health Services Management Training Centre to complement the national viewpoints with observations by international experts. The expert interviews provided an opportunity to map the views and experiences of international organisations; thus, the country-level information was completed and triangulated with international-level information.

The interviewees were international experts whose expertise is linked to international and/or European projects and data collections, identified during an earlier literature review. Experts from international organisations (WHO, OECD) were selected based on their fields of expertise and earlier contributions to the present topic at the international level and were invited to participate in the interviews. In total, six international experts were identified and invited to participate. All of these experts were interviewed via telephone or in person. The interviews lasted about 40–50 min and covered national and international health workforce data collection problems and opportunities. Headcount and FTE issues were also discussed in detail (see interview guide in the Additional file 2). A summary of each interview was prepared by the interviewers, and the interviewees reviewed and confirmed the accuracy of the summaries. These summaries were then thematically analysed by the research team in parallel to the survey analysis in order to obtain supplementary information for international viewpoints. No qualitative analytical software was used for performing the analysis.

## Results

Of the 14 countries, Belgium, Germany, Hungary and the United Kingdom indicated that they reported FTE to the JQ and that they calculate and use FTE data for national planning purposes as well. Each FTE calculation formula used by these four countries matches one of the OECD recommendations for FTE calculation. The other 10 countries investigated do not use FTE data for national purposes. Seven of these 10 countries tended to provide FTE data to the JQ using special calculations and/or estimation methods for converting headcount data to FTE data. Cyprus, Greece and Iceland on the other hand pointed out that they did not possess any FTE calculation methods for JQ reporting. In total, 11 countries reported FTE data for the JQ data collection about all five sectoral professions.

Table 3 shows the variety of different national calculation methods used when reporting FTE data to the JQ in terms of the five sectoral professions. These examples demonstrate the great variety of methods for calculating FTE across EU countries. Not surprisingly, there is no common FTE calculation method or formula. All of the participating countries calculate or estimate FTE according to their national practice, in their own way. What is most likely is that the countries have different traditions and working patterns for their labour market and operate with different data systems, which may explain the differences in FTE calculations. This calculation and estimation diversity can lead to misinterpretations, resulting in biases in international comparisons. Some of the calculation and estimation methods that differ significantly are (1) the definition of full-time or part-time workers (e.g. using 0.5 or 0.6); (2) the baseline calculation period (weekly working hours or 2-week period); (3) gender; or (4) setting-related FTE. It is important to emphasise that Spain, for example, counts male and female health professionals separately; thus, they have different values for FTE. These results already reveal differences in the calculations, which can lead to inaccurate comparisons and conclusions based solely on the FTE indicator.

**Table 3:** National FTE calculation/estimation methods

Belgium	Medical doctors, dentists and nurses: calculation from actual number of working hours.
	Independent professionals: calculation based on the median income of the 45–54 age group (and comparison to it). Working groups: experts’ estimation based on expert knowledge and data.
Finland	FTE = 1 × headcount of full-time persons, 0.6 × head count of part-time persons and 0 × head count of persons on leave.
	Rough estimations are based on municipal data. The estimate of part-time is 60 % and was estimated from samples a long time ago.
Germany	The number of FTE is calculated by adding the number of full-time and an appropriate proportion of part-time employees together. FTE are measured by the number of hours of a standard labour contract.
Hungary	The number of part-time workers is converted into full-time equivalent in the following way: the actually performed weekly working hours are divided by the weekly compulsory labour time (40 h or 36 h) as stipulated by the law for individual jobs. The value of the full-time equivalent can be a whole number and one decimal.
Ireland	The whole-time equivalent (WTE) calculation is performed on the basis of the number of hours worked in the 2-week period in the prior month and divided by the standard number of hours worked in a normal 2-week period. This is calculated only for the JQ data collection, and there is no FTE data for other professions.
Italy	The data concerning full-time and part-time (less than 50 % and more than 50 %) is available only for the public sector.
Poland	The headcount is conducted on the basis of the main place of employment. Professionals pursuing the sectoral professions must provide their registers with information on all the places where they are currently starting employment. If they work in more than one place, they have to provide information on all of the other places as well.
Portugal	The Health System Central Administration (ACSS_PT) has data from the National Health System on headcount and full-time equivalent (FTE). FTE is calculated from the actual number of hours/week that a health professional works as a percentage of FTE (35H) or FTE (40H), depending on what is established in the specific contract.
Spain	Simple calculation method
	FTE (man) = 0.917 × male headcount
	FTE (female) = 0.826 × female headcount
	Holidays and other work exemptions (illness, teaching, research, etc.) are considered, so that 1 male headcount is not equivalent to 1 FTE.
The Netherlands	For salaried professionals, headcount and FTE are available. For self-employed professionals, only headcount is available from Statistics Netherlands. FTE is available from other sources, and most often, data on FTE for self-employed professionals can be found in surveys. The Advisory Committee of Medical Manpower Planning (ACMMP) has conducted surveys among self-employed doctors to self-report FTE as an estimate for self-employed doctors.
The United Kingdom	In one of the four countries of the United Kingdom, England, FTE is defined (in the workforce census bulletin) as “a standardised measure of the workload of an employee”. An FTE of 1.0 means that a person is equivalent to a full-time worker; an FTE of 0.5 signals that the worker is half (part) time. FTE is the full-time equivalent and is based on the proportion of time staff work in a role.

This finding on diversity was confirmed by the experts; nonetheless, FTE may still serve as a comparative tool for HWF monitoring and planning (e.g. working patterns, HWF supply). The experts also emphasised that difficulties may occur in data collection, data availability and the lack of standard, universally accepted calculation methods for the indicator (e.g. normal working hours may vary from 35 h to 55 h per week depending on the profession such as nurses or doctors, as well as between systems within a country such as public or private). These various factors constitute a significant burden that may weaken the robustness of this measurement category.

In terms of expert opinions, aggregated FTE data for entire professions do not support performing international comparisons and benchmarking for HWF planners, because for HWF planning, personal-level FTE data would be needed. Such aggregated data does not provide a detailed level of information for refined future comparisons. According to the experts, following up after individual data could ensure much greater value for analyses and their subsequent use for HWF planning and forecasting.

In summary, many of the experts agreed that the activity of healthcare professionals is a fundamental factor in HWF monitoring and planning, which should also be measured in FTE in addition to headcount. Due to its significant relevance, FTE has a special usefulness for HWF planning. Experts highlighted the increasing importance of FTE due to the latest trends, such as social changes and gender changes of the HWF (i.e. the part-time work option, although it should be added that gender is only significantly considered in Spain).

## Discussion

The diversity of the FTE calculation method is of concern, since remarkable shortages of health professionals exist in the healthcare sector [2], and there is no internationally comparable data set on the capacity of the health systems. National and international monitoring processes need to be developed in order to support informed health policy decision-making. The calculation and utilisation of headcount and FTE data could support the understanding of the current HWF situation, and the continuous monitoring of headcount and FTE could contribute to better informed policy-making that would influence the future workforce. Additionally, it could contribute to human resource management and development, strategic health workforce monitoring, and planning at different levels (e.g. regional, national).

The OECD-recommended formulas might reduce the diversity of FTE calculation methods, since they are accepted as comparable methods, and provide support for those countries which have no FTE data available. The OECD-recommended formulas can support countries in improving their data calculations in regard to FTE, thus providing the JQ with more complete international reporting. FTE estimates could support a deeper understanding of the domestic HWF and enhance national-level health workforce monitoring and planning activities. The importance of FTE is increasing due to the latest trends, such as social and gender changes in the HWF, and the expectations for a more balanced lifestyle from new generations [14]. Therefore, FTE could provide a better overview of real HWF activity, which is essential for planning purposes.

As Dussault et al. stated, only flexible health systems and health workforces can adapt efficiently to continuous demographical, epidemiological and social/societal changes [3], and it is particularly relevant to consider the expected challenges of the future [14]. Consequently, the role of FTE is obviously of increasing importance in national and international HWF monitoring, forecasting and planning. The difficulties in internationally comparing data are already known [6]. Our results improved and clarified the question in regard to FTE data collections and raised awareness with respect to different national data collections, estimations and the issue of international comparability.

In addition to FTE, there are further limitation issues in regard to the international HWF data collection process. Other important issues requiring attention are the exact definitions of different professional categories (e.g. nurses) and activity categories, which are also collected by the JQ [11]. These issues are significant for national-level HWF monitoring and are also covered by the Joint Action [9].

Our study has one important limitation: the questionnaire cannot differentiate between the FTE calculations used between professions. As a result, this limitation has to be considered in the discussion of the results. This is an area that should be addressed in research conducted in the future.

## Conclusions

FTE and headcount are significant factors in health workforce planning and monitoring; therefore, national data collections should pay significant attention to collecting this data and calculating the appropriate indicators. FTE and headcount both reflect the health workforce supply from different angles; therefore, they complement each other. FTE contributes to the assessment of the actual working hours of the national workforce and describes the working patterns of the workforce, i.e. the number of hours expected across a working week. FTE does not, however, demonstrate the time health professionals spend with patient- and non-patient-related activities. Most of the investigated countries do not use FTE data for national purposes, and deficiencies exist in data reporting as well. Nevertheless, even with such a constant element of error, FTE may be relevant for highlighting trends in the employment activities of health professionals. Raising awareness and building capacity for FTE calculations could support health policy decision-making in health workforce planning and monitoring. A national FTE could serve as a call to action for HWF planners due to the lack of matching international FTE data.

At the international level, it is beneficial to monitor the trends and numbers regarding human resources and time worked in order to track changes and recognise the actual situation across the EU. Since most countries do not follow OECD recommendations for FTE calculation, comparisons between the FTE data of different health systems may be misleading. Comparability could improve if countries were to use standardised calculation methods and formulas.

Comparing FTE at the international level—even if for well-selected subgroups of the health workforce—requires detailed metadata. Considering the complexities and differences in measuring FTE in different countries, FTE may not be regarded as a feasible data collection category across various health professions and sectors at the EU level in the near future. For the moment, the exchange of information and mutual assistance in developing the capacity to apply common methodology could be a first step towards standardised data collection. Nevertheless, the exchange of selected FTE information between countries may be feasible and may significantly contribute to HWF planning processes and better informed policy-making.

## Additional files

Additional file 1: The survey question about data on headcount and on full-time equivalent. (DOCX 12 kb) Additional file 2: WP4 interview guide for semi-structured expert interviews. (DOCX 47 kb)

## References

[1] Sermeus W, Bruyneel L. Investing in Europe’s health workforce of tomorrow: scope for innovation and collaboration. WHO European Observatory on Health Systems and Policies; 2010. https://www.enpam.it/wp-content/uploads/Report-PD-Leuven-FINAL.pdf.

[2] WHO (2006). Working together for health: the World Health Report 2006.

[3] Dussault G, Buchan J, Sermeus W, Padaiga Z. Assessing the future health workforce needs. World Health Organisation European Observatory on Health Systems ans Policies; 2010. http://www.euro.who.int/__data/assets/pdf_file/0019/124417/e94295.pdf?ua=1.

[4] Malgieri A, Michelutti P, Hoegaerden MV (2015). Handbook on health workforce planning methodologies across EU countries.

[5] Buchan J, Wismar M, Glinos IA, Bremner. Health professional mobility in a changing Europe. WHO European Observatory in Health Systems and Policies; 2014. http://www.euro.who.int/en/about-us/partners/observatory/publications/studies/health-professional-mobilityin-a-changing-europe.-new-dynamics,-mobile-individuals-and-diverse-responses. 29144691

[6] Matrix Insight Ltd, Centre for Workforce Intelligence (2012). EU-level collaboration on forecasting health workforce needs, workforce planning and health workforce trends—a feasibility study.

[7] JA on Health Workforce Planning and Forecasting (2014). JA Health Workforce Planning and Forecasting D.051—minimum planning data requirements for health workforce planning.

[8] Commission; E, Commission E (2003). 2003/88/EC working time directive. 2003/88/EC.

[9] Aszalos Z, Eke E, Kovács E, Kovács R, Cserháti Z, Girasek E, Wéber A, Hoegaerden MV (2015). Joint action on health workforce planning and forecasting—WP4 Terminology gap analysis D041.

[10] Diallo K, Zurn P, Gupta N, Dal Poz M (2003). Monitoring and evaluation of human resources for health: an international perspective. Hum Resour Health.

[11] OECD, Eurostat, WHO-Europe (2014). Joint data collection on non-monetary health care statistics—guideline for completing the OECD/Eurotat/WHO-Europe Questionnaire 2015.

[12] OECD. OECD statistics. Paris, France: OECD; 2015.

[13] European Commission, Commission E (2005). Directive on the recognition of professional qualifications. 2005/36/EC.

[14] Centre for Workforce Intelligence. Shape of the medical workforce: starting the debate on the future consultant workforce. London: Center for Workforce Intelligence; 2012. http://www.cfwi.org.uk/publications/leaders-reportshape-of-the-medical-workforce/@@publication-detail.

