# Supplementary material for: Headcount and FTE data in the European health workforce monitoring and planning process
Source: Hum Resour Health. 2016 Jul 16;14:42. doi: 10.1186/s12960-016-0139-2 (PMC4947240; doi:10.1186/s12960-016-0139-2)
Supplement: Additional file 1: — The survey question about data on headcount and on full-time equivalent. (DOCX 12 kb) [file 12960_2016_139_MOESM1_ESM.docx]

**Additional File 1.**

**1.A.4. HWF data on headcount and on full-time equivalent**

| How do you collect headcount and full-time equivalent data in your country for the 5 sectoral professions? Please indicate how you calculate FTEs. |
| --- |
| \| HWF data only in headcount. \|  \| \| --- \| --- \| \| HWF data only in full-time equivalent (FTE). \|  \| \| HWF data both in headcount and FTE. \|  \| \| Please indicate how do you calculate FTE. \|  \|   *(Please add your comments here.)* |
