# Supplementary material for: Headcount and FTE data in the European health workforce monitoring and planning process
Source: Hum Resour Health. 2016 Jul 16;14:42. doi: 10.1186/s12960-016-0139-2 (PMC4947240; doi:10.1186/s12960-016-0139-2)
Supplement: Additional file 2: — WP4 interview guide for semi-structured expert interviews. (DOCX 47 kb) [file 12960_2016_139_MOESM2_ESM.docx]

**WP4 Interview guide for semi-structured expert interviews**

Interviewee/Affiliation:

Date (Length) of the interview:

Interviewer:

Terminology issues concerning JQ

Introduction: WP4 activities focus on several issues, and one of the main crucial issues deals with terminology/data source gaps and the mapping of the existing difficulties in definition-related problems in MSs when reporting to Joint Questionnaire.

Objective: The aim of the present interview is to see what experts think of the existing terminology/definitions/data content, and what experts consider about the accuracy, accessibility, timeliness and comparability of data based on terminology/definitions; and their use, understanding and problematic points/difficulties in different MSs.

**1) What are your experiences about the existing terminology/definitions used in different MSs compared to/in reflection of JQ categories?**

____________________________________________________________________________________________________________________________________________________________________

What is your opinion: Is there a clear and common terminology/definition on EU level? Are they mostly based on qualification or occupation data? What do you think about these categories? What problems do you see in having a comparable data on these? Do/can MSs collect and report these? Are JQ categories feasible to collect in MSs? Shall we keep these three categories (licensed to practice, professionally active, practicing)? Would it be sufficient to collect fewer categories? Or shall we need more/more detailed categories? How clearly are these categories divided? Is there a need for new EU terminology? If yes, then how shall we elaborate a new EU terminology?

If professionally active/practicing category problems are mentioned: How can we measure activity-direct patient contact? How can we measure the number of professionals without direct patient contact?

If headcount and FTE issues are mentioned: What is more important in HWF monitoring/planning/forecasting: HC or FTE? How shall be FTE calculated? Do we need a standard formula for it? Shall we make a difference on employment: salaried professionals and self-employment?

**2) What is your opinion about the practical gains of using ISCO codes in the context of JQ data collection, taking into account its content and original purpose?**

____________________________________________________________________________________________________________________________________________________________________

How do ISCO Codes support the harmonization of terminology? How does the Directive support the harmonization of terminology? Should ISCO be updated?

If ESCO is mentioned: What is your opinion about ESCO? Can this initiative balance terminology gaps in order to have more real-life data? Shall we combine definition with tasks completed under one profession?

Nurses and midwives: How do you see the overlaps between these categories?

**3) How do you see the activities of National Focal Points?**

____________________________________________________________________________________________________________________________________________________________________

What mechanisms could support this information flow? Shall we/How shall we facilitate international information and data flow?

**4) What actions need to be done in order to develop reliable and valid databases in EU?**

____________________________________________________________________________________________________________________________________________________________________

What shall be done in order to have/gain the currently non-available data? What practical recommendations and steps do you see in developing EU data collection systems, particularly the JQ?

**5) What shall be the main purpose of JQ data collection and database?**

____________________________________________________________________________________________________________________________________________________________________

How could JQ support monitoring/ benchmarking/planning? What should be the main purpose of this data? How could MSs benefit from the JQ data? What HWF data (e.g. FTE or gender) should be collected at international level? Should national level experts using HWF data for monitoring and planning purposes be made aware of the potential of JQ data? If yes, what solutions do you recommend?

Is there anything we have not touched during this conversation and you think is worth mentioning?
